# Screening of Marine Bioactive Antimicrobial Compounds for Plant Pathogens

**DOI:** 10.3390/md19020069

**Published:** 2021-01-28

**Authors:** Xiaohui Li, Hejing Zhao, Xiaolin Chen

**Affiliations:** 1 College of Food and Pharmaceutical Sciences, Ningbo University, Ningbo 315832, China; lixiaohui@nbu.edu.cn (X.L.); 196003457@nbu.edu.cn (H.Z.); 2 State Key Laboratory of Agricultural Microbiology and Provincial Hubei Key Laboratory of Plant Pathology, College of Plant Science and Technology, Huazhong Agricultural University, Wuhan 430070, China

**Keywords:** marine natural products, plant pathogens, bioactive substances, chemical control, antimicrobial mechanism

## Abstract

Plant diseases have been threatening food production. Controlling plant pathogens has become an important strategy to ensure food security. Although chemical control is an effective disease control strategy, its application is limited by many problems, such as environmental impact and pathogen resistance. In order to overcome these problems, it is necessary to develop more chemical reagents with new functional mechanisms. Due to their special living environment, marine organisms have produced a variety of bioactive compounds with novel structures, which have the potential to develop new fungicides. In the past two decades, screening marine bioactive compounds to inhibit plant pathogens has been a hot topic. In this review, we summarize the screening methods of marine active substances from plant pathogens, the identification of marine active substances from different sources, and the structure and antibacterial mechanism of marine active natural products. Finally, the application prospect of marine bioactive substances in plant disease control was prospected.

## 1. Introduction

According to the world food program, by the end of 2020, the number of people facing serious food security will increase to 270 million, and 25 countries will face population hunger [1]. The fundamental method to solve the problem of food shortage is to increase crop yield, which is seriously affected by plant diseases. Therefore, effective control of crop diseases is one of the important ways to solve the world food shortage. Traditionally, agricultural synthetic chemicals, including fungicides and bactericides, have been widely used in the control of plant pathogens. Although most of the chemical agents have the characteristics of high efficiency and persistence [2,3], many of them are toxic and harmful to the environment, sometimes leading to high resistance of pathogens (due to the evolution of pathogenic bacteria, pathogenic mutation, etc.) [4,5,6,7]. Therefore, the development of biological microbicides with the advantages of low toxicity, high efficiency, and lower induction of resistance are more acceptable and welcome in the present.

Biological fungicides are bioactive substances produced by living organisms or synthetic chemicals derived from natural products with a similar structure as lead compounds [8,9]. Previously, biological fungicides were mainly developed from terrestrial organisms [10,11]. However, after a long period of screening in recent years, the terrestrial biological resources are extremely reduced, and people pay more attention to the marine organisms, which account for 71% of the earth’s biological resources. Marine biological resources are extremely rich, and the special marine environment makes it possible for marine organisms to produce different natural active substances from terrestrial organisms [12,13,14]. The structures and pharmacological effects of these marine-derived active substances are usually unmatched by those derived from the terrestrial organisms. Thus far, scientists have isolated numerous natural products with antitumor, antibacterial, and other biological activities from marine organisms [12,15,16,17,18,19]. Modern pharmacological studies also demonstrate that many marine biological metabolites show good effects on killing phytopathogenic bacteria, which possibly can be used for developing biological fungicides [20,21,22,23,24]. In fact, at the present, marine organisms have become an important resource for developing new biological fungicides to control plant diseases. In this review, we summarize the research progress of marine natural compounds, including the culture of marine microorganisms, the isolation and extraction of natural compounds, activity screening, and antibacterial mechanism.

## 2. Methodologies of Screening Marine Antimicrobial Substances

**Figure 1:**
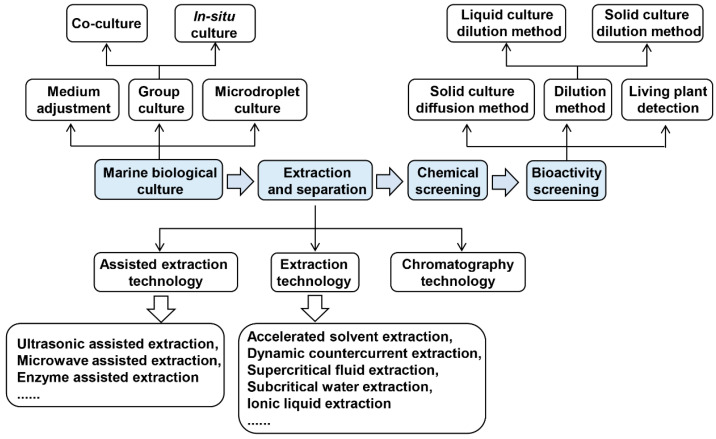
Research flow for isolation of marine natural compounds against plant pathogens.

With the rapid development of screening technology for marine antimicrobial substances, the screening process has been continuously promoted with the expansion of the scope of application [25]. The culture methods of marine microorganisms, isolation and extraction of natural compounds and activity screening will be described in the following chapters. Figure 1 shows the research process of isolation of marine natural compounds for plant pathogens.

### 2.1. Cultivation of Marine Organisms

Based on their cultural characteristics, the marine organisms are classified into two categories, one is cultivable organisms, and the other is non-culturable organisms. Cultivable organisms can be cultivated artificially, while non-culturable organisms cannot [26,27,28]. For some large-sized marine organisms, their biomass is sufficient enough to extract natural products for testing, whether they can be cultured or not is not important. Other marine organisms, especially marine microorganisms, are usually small-sized with low biomass [25]. To obtain a certain amount of natural products, it is necessary to expand the culture of the microorganisms, which requires that the marine microorganisms can be cultivated. However, up to now, the cultivable microorganisms in the ocean only account for 1% of the total microorganisms [26,27,28]. The reason why marine microorganisms are not cultivated is not only because of low abundance, but also because some microorganisms need to grow under special conditions, such as temperature, pH, and nutritional conditions [26,27,28]. In addition, some living microorganisms enter a dormant state in order to adapt to adverse environmental conditions. When the external conditions become more favorable or induced by growth suitable signals, these microorganisms can recover and grow again [29]. Therefore, there is a process of signal exchange between microorganisms and the environment. Once this signal exchange is interrupted, the microorganisms may not grow. This is also one of the most important reasons why marine microorganisms cannot be cultivated. In response to this problem, scientists developed some strategies to separate and culture these uncultivated microorganisms.

#### 2.1.1. Medium Optimization

First, bacteria and fungi need water, a carbon source, a nitrogen source, and inorganic salt to survive [30]. Bacteria have different specific carbon and nitrogen needs according to the difference between autotrophic and heterotrophic bacteria. Autotrophic bacteria can usually convert inorganic carbon into organic carbon. In this case (photo autotrophic, chemical autotrophic), external energy, such as light energy or chemical energy, is usually required. Heterotrophic bacteria must absorb energy from organic carbon sources, such as carbohydrates, ethanol, acetic acid, etc., and may or may not use inorganic nitrogen sources. Heterotrophic bacteria can also be classified as aerobic, facultative aerobic, or anaerobic, depending on whether the organic carbon source is oxidized to convert energy through oxygen oxidation or other oxidation pathways [31]. Fungi basically live a heterotrophic life, the carbon source they need to grow is usually comprised of carbohydrates, and the nitrogen source is usually comprised of amino acids or proteins; some fungi can undergo anaerobic fermentation, but most of them are aerobic or facultative aerobic types. Many fungi grow slower than bacteria, but fungi are more tolerant of high salt than bacteria, and are more suitable for growing in a wider temperature [32]. In short, different types of microorganisms require distinct environments and nutrients for their growth [33]. Therefore, it is necessary to explore the proper environment and nutrients demands for one specific microorganism, which can be used for developing medium formulations suitable for it.

Studies have shown that some "uncultivated" microorganisms can be grown through a modified medium [34,35]. For example, using unconventional electron donors, electron acceptors and carbon sources can effectively cultivate many new microorganisms. Santini et al. isolated autotrophic bacteria (*NT-26*) from the Aodaliya gold mine with arsenous acid as an electron donor, oxygen as a receptor, and carbon dioxide as the sole carbon source [36]. *NT-26* belongs to a branch of *Rhizobium* of α-Proteobacteria *Agrobacterium* [36]. Additionally, because the specific environment of microorganisms can catalyze new redox reactions, some unconventional improved media can be used to isolate many novel microorganisms based on this feature. Using a refined medium containing only hydrocarbon/carbon energy and nitrate, iron salt or sulfate as electron acceptors, bacteria that degrade hydrocarbons were identified [37]. The quality of the carbon source is a key factor in determining culture efficiency. For example, a yellow-producing bacterium, TRM1, identified from tomato soil microorganisms can only use D-mannose as the carbon source, suggesting that D-mannose can be used as the sole carbon source to enrich the population of TRM1 [38].

#### 2.1.2. Co-Cultivation

Second, in nature, some microorganisms need to use specific bio-substances or collaborate with other microorganisms for resource utilization [39]. For example, Crocetti et al. cultivated anaerobic thermophilic glutamic acid-degrading microorganisms from anaerobic sludge (mixed microbial communities) using the culture method of dialysis membrane reactor [40]. Nichols et al. used the helper strain MSC105 to successfully cultivate a new genus, Psychobacterium MSC33, from standard culture media. MSC105 can provide the necessary growth factors for the growth of MSC33 [41]. Tanaka et al. isolated a wide variety of novel microbes using a duckweed-microbe co-cultivation approach [42]. These results all indicate that in the microbial flora of the natural environment, the interaction between microorganisms is crucial to the survival of the colony, and they can cooperate through the exchange of metabolites and signal molecules. Therefore, for some microorganisms that are more difficult to cultivate, it can be tried to co-culture with other microorganisms.

#### 2.1.3. In Situ Cultivation

Third, simulating the natural growth environment of microorganisms is a basic strategy to isolate and cultivate the oligotrophic marine microbe. The microorganisms can be placed in a standard culture medium covered with a membrane, by which the signal molecules can pass through but the microorganisms cannot. Then the culture device is placed in the original environment where the microorganisms are activated by the signals for growth. The rationality of this method is that some natural growth factors can be diffused into the cultivation medium through the membrane, thereby activating the growth of microorganisms for cultivation [43]. Nichols et al. designed a separation chip (Ichip) to isolate and culture microorganisms. In this method, the central plate was immersed in the testing microorganism suspension for cultivation and was then transferred to the agar medium. Then the medium is fixed by cooling, and both sides of the plate are attached with films and separated by squeezing [27]. Around 40% of the marine microbial species and 50% of the soil microbial species were isolated by this chip. Among them, 62 (28.3%) of the seawater samples and 86 (28.7%) of the soil samples were pure cultured microorganisms, and the similarity of the 16S rRNA gene was less than 95% [27]. This method can be used to isolate and culture a large number of microorganisms with high diversity [44].

#### 2.1.4. Microdroplets Cultivation

Zengler et al. designed a smart microdroplet-based method to isolate microorganisms. In this method, the microorganisms embedded in a gel microtiter plate and cultured in a low-nutrient flow medium. They were subsequently detected on the microtiter plate used flow cytometry to determine the number of microclonies [45]. This is an open-flow system, in which the metabolites and signal molecules between microorganisms can diffuse in the gel gap and be used by other bacteria. This has many similarities with the natural environment so that the types of newly cultivated microorganisms have greatly increased. This method can be applied in environments such as soil and seawater, and more than 10,000 bacteria and fungi were successfully isolated from each environmental community [45]. Using the microdroplet-based technique, single cells were isolated and cultured from the soil microbial community enriched in polycyclic aromatic hydrocarbons, and four mycobacterial isolates and one previously unknown fluoranthene-degrading *Bacillus* species was isolated [46]. Compared with the previous microdroplet-based method, this method does not require expensive automated equipment to generate microdroplets, and the operation is simpler and easier.

### 2.2. Extraction and Separation of Marine Natural Products

The extraction and separation technique are another important factor for mining of marine natural products. However, the traditional technology of extraction and separation of active ingredients from natural products is inefficient, glossy and complicated. For example, the traditional solvent extraction method is solvent-consuming and time-consuming, the sublimation method and steam distillation method have special requirements for the composition, the two-phase extraction is easy to emulsify and the separation time is long [47]. Therefore, new extraction and separation technologies were developed to overcome these drawbacks. In the following part, we summarized these new extraction and separation technologies.

#### 2.2.1. Auxiliary Extraction Technique

##### Ultrasound-Assisted Extraction (UAE)

Ultrasound can effectively destroy the microbial cell wall, increase the penetration of the solvent, and achieve effective extraction of the compound. Compared with traditional organic solvent extraction methods, the UAE technique has no obvious heating process, so it does not change the molecular structure of the compound [48]. Quiroz et al. used the UAE technique to obtain antibacterial and antioxidant extracts from annatto seeds and determined the most suitable extraction conditions including an ultrasonic treatment for 20 min [49]. From the seaweed *Nizamuddinia zanardinii*, Alboofetileh et al. extracted sulfated polysaccharides through the UAE method [50]. The UAE technique is relatively simple, but lower in efficiency compared with other methods.

##### Microwave-Assisted Extraction (MAE)

MAE improves the heat and mass transfer performance of the extraction process by using the characteristics of microwave. It is easy for selection, save the operation time, the solvent consumption low, and the yield is high with effective ingredients, which is widely used for extracting sugars, saponins and polyphenols [51]. For example, through the MAE technique, the phlorotannins was successfully extracted from *Fucus vesiculosus* [51]. Sebastian et al. also used MAE method to separate and extract chitinase from the fungus *Rhizopus oryzae* NRRL1526, with a high final chitinase yield [52].

##### Enzyme-Assisted Extraction (EAE)

As a natural catalyst, enzymes can decompose the microbial cell walls under normal temperature and pressure. Biological enzymatic extraction technique utilizes the catalytic properties of enzymes. According to the nature of the active ingredients to be extracted, we can select the corresponding enzymes to destroy the cell wall structure for extraction [53]. Using the EAE method, Nguyen et al. successfully extracted a fucoidan compound from two brown algae [54]. Habeebullah et al. used different enzymes to extract active substances from six different brown seaweeds and obtained a number of compounds with anti-fungal or anti-bacterial activities [55]. This report shows that the enzyme-assisted extraction technique can be used to prepare seaweed extracts with specific biological activities.

#### 2.2.2. Extraction Technique

##### Accelerated Solvent Extraction (ASE)

The ASE technique can extract solid or semi-solid samples under higher temperature (50–200 °C) and higher pressure (10.3–20.6 MPa). This technique has the advantages of saving time and solvent [56]. Johnson et al. respectively used standard solvent separation (SSP) and ASE methods to extract 12 active substances from sponges. The result demonstrated that the extraction efficiency of the ASE method was three times higher than that of the SSP method, and the extraction effect of 2 h with ASE was equivalent to that of 80 h with the SSP method [57].

##### Dynamic Countercurrent Extraction (DCE)

DCE technique refers to the process that raw materials and solvent are added from both ends of the extraction device, and under the action of mechanical force and gravity, the solvent penetrates into the raw materials to increase the concentration [58]. Yuan et al. used this method to separate and extract bafilomycin A1 (**1**) from the marine *Streptomyces lohii* fermentation, the recovery rate is as high as 95% [59]. From the marine fungus Alternaria alternate HK-25, He et al. isolated five diketopiperazines by using DCE with high recovery rates (>94%) [60].

##### Supercritical Fluid Extraction (SFE)

The SFE technique take the advantages of supercritical fluids with high permeability and solubility to dissolve solutes in the fluid at higher pressures. Through pressure and temperature adjustment of the supercritical fluid, SFE can separate different components [61]. Supercritical fluids have strong penetrating power similar to gases and greater density and solubility similar to liquids. CO_2_ is a common supercritical fluid and is suitable for extracting some low-polarity substances, the extraction efficiency for medium and high polar substances is often not ideal. If some polar modifiers are added to it, this situation can be improved. The supercritical CO_2_ extraction technique has the characteristics of no solvent residues and continuous operation, which is suitable for large-scale production [62]. Messina et al. have successfully used the SFE technique to extract anti-oxidant and pro-apoptotic astaxanthin (**2**) from Parapeneus longirostris [63]. From the Spirulina Cyanobacterium Arthrospira, Yang et al. successfully extract γ-linolenic acid by using SFE [64].

##### Subcritical Water Extraction (SWE)

Compared with supercritical extraction, SWE has a lower working pressure, which reduces the investment cost of high-pressure equipment. It also has the advantages of large processing capacity and no solvent residue, which is suitable for process production. The SWE technique can extract non-polar, weakly polar, and strong polar compounds by controlling temperature and pressure conditions [65]. Pangestuti et al. used the SWE system to hydrolyze two mangroves. In order to enhance the best utilitarian materials, three temperature conditions with 40 °C augmentations were applied [66]. Alboofetileh et al. isolated the fucoidan, a sulfated polysaccharide from N. zanardinii by using an upgraded SWE with a reaction surface strategy improved extraction conditions. This method optimized the reaction conditions by setting extraction time as 29 min, temperature as 150 °C, as well as material-to-water proportion to 21 g/mL, which resulted in a much higher yield (25.98%) [67].

##### Ionic Liquid Extraction (ILE)

Ionic liquid exists as the liquid at room temperature or near room temperature. Compared with organic solvents, it has the characteristics of not volatile, good solubility, and adjustable structure. It is considered to be a new green solvent that can replace traditional solvents. Ionic liquid, inorganic salt, and water form an efficient and gentle new green separation system, which has the advantages of short phase separation time, low viscosity, not easy to emulsify during extraction, and ionic liquid can be recycled [68]. Liu et al. used the ionic liquid to pre-treat *Haematococcus pluvialis* to destroy the cell wall, thereby improving the extraction efficiency of astaxanthin [69]. The ILE technique also successfully extracted an extracellular protein from the biofilm matrix of the anammox marine bacterium [70].

#### 2.2.3. Chromatography Technique

Chromatography is an important method for separating natural compounds. It uses the different distribution coefficients of different substances in different phases to elute with the mixture in the mobile phase and the stationary phase. Different substances in the mixture will be eluted at different speeds. It moves along the stationary phase and finally achieves the separation effect. It has a very wide range of applications in natural products and other fields [71]. According to the separation mechanism of substances, chromatography can be divided into the following three categories: 

##### Separation Based on Distribution Ratio of the Substance

The separation is achieved by using the difference in solubility of the components to be separated between the stationary phase and the mobile phase. The stationary phase of partition chromatography is generally a liquid solvent, which is distributed on the surface of the chromatographic column or support by means of coating, bonding, and adsorption. The process of partition chromatography is essentially a process in which the component molecules continuously reach equilibrium between the stationary phase and the mobile phase. The following describes several commonly used such chromatographic methods.

High-speed countercurrent chromatography (HSCCC) technique is based on liquid-liquid partition, which uses the directionality of the spiral tube combined with the high-speed planetary motion to realize efficient distribution in the immiscible two-phase solvent system. With the continuous injection of the mobile phase, different components can be separated from small to large according to the partition coefficient [72]. Compared with other chromatographic separation technologies, HSCCC does not use a solid phase carrier, which avoids the occurrence of adsorption pollution, denaturation, and deactivation caused by the use of carriers in liquid-solid chromatography. It has a wide range of applications, simple operation, high recovery rate, etc., and realize micrograms and microliters scale analysis [73]. The anti-microbial polyketide JBIR-99 (**3**) was successfully purified by HSCCC, from a marine fungus Meyerozyma guilliermondii [74]. Another compound phytosterols was also isolated by HSCCC from Sargassum horneri, a brown seaweed [75].

Reversed phase chromatography (RPC) is another chromatographic method that uses a non-polar stationary phase for separation. In RPC, polar compounds are eluted first, while non-polar compounds are retained due to their affinity of the reversed-phase surface [76]. At present, RPC accounts for the vast majority in the analysis of liquid chromatography. Using the reversed-phase high performance liquid chromatography, cyclic lipopeptides, fengycins, were separated from the marine Bacillus subtilis [77]. Fengycins have the activity to kill the plant pathogenic fungus Magnaporthe oryzae [77]. Using a similar technique, another compound, plipastatin A1, was isolated from marine B. amyloliquefaciens SH-B74 [78]. Plipastatin A1 can inhibit gray mold [78].

##### Separation based on Adsorption of the Substance

For this type of chromatography, the separation of the mixture is achieved by using the difference in the adsorption capacity of the substance molecules in the stationary phase adsorption. During the separation process, the mobile phase molecules and the substance molecules compete for the adsorption center of the stationary phase. In this strategy, the macroporous adsorption resin method is the most commonly used. The macroporous adsorption resin is mainly made of styrene and divinylbenzene, which are polymerized by adding a certain proportion of porogen in a 0.5% gelatin solution. The adsorption effect of macroporous resin relies on the van der Waals attraction between it and the adsorbents, and work through physical adsorption through its huge specific surface. During separation, organic compounds can pass through a certain solvent according to the adsorption force and molecular weight [79]. Through the macroporous adsorption resin adsorption method, Chen et al. separated the anti-tobacco mosaic virus (TMV) ε-Poly-l-lysine compounds from *Streptomyces ahygroscopicus* [80], and He et al. separated the anti-gray mold compound tetramycin A from ZC-G-5 [81].

##### Separation Based on Molecular Size of the Substance

For this type of chromatography, the components to be separated will enter or not enter the pores of the stationary phase gel depending on the molecular weight. The molecules which cannot enter the pores of the gel will quickly elute with the mobile phase and can enter the gel. The molecules in the pore need to be washed for a long time to flow out from the stationary phase, so as to achieve the component separation based on the molecular weight difference. Adjusting the crosslinking degree of the gel used in the stationary phase can adjust the size of the gel pore; changing the solvent composition of the mobile phase will change the swelling state of the gel, and then change the pore size to obtain different separation effects. The currently commonly used method is Sephadex chromatography, a chromatographic method that uses Sephadex as the chromatographic matrix, which can be used to separate proteins and other compounds with smaller molecular weights [82]. This method has been successfully used to separate polyhydroxy steroid and tricyclic diterpenoid, which can inhibit gray mold, from the marine fungus [83]. Another compound, benzopyranone that inhibits the growth of *Alternaria brassicicola*, was also identified from the marine bacterium *Alternaria* sp. (P8) [84].

### 2.3. Chemical Screening

Chemical screening is the screening of new chemical structures without considering their biological activity. The isolated compounds were obtained according to the previous methods. Then the optical methods (UV/MS/NMR) or thin layer chromatography (TLC) methods were used to analyze the properties of these compounds. UV and TLC methods are relatively old methods [85,86], and currently mass spectrometry (MS) and nuclear magnetic resonance (NMR) methods are usually used to analyze the properties of compounds.

MS is an analytical method for measuring the mass-to-charge ratio (mass-to-charge ratio) of ions [87]. The basic principle is to ionize each component in the sample in the ion source to generate charged ions with different charge-to-mass ratios, accelerating the action of the electric field to form an ion beam and enter the mass analyzer [87]. In the mass analyzer, the electric field and magnetic field are used to make the opposite velocity dispersion, and they are respectively focused to obtain a mass spectrum to determine its mass. The relative molecular mass, molecular formula and structural unit of an unknown compound can be obtained by mass spectrometry analysis.

Nuclear magnetic resonance spectroscopy is a powerful analytical method for studying the structure of matter. The main principle is that in a strong magnetic field, the nucleus and electron energy of certain elements have their own magnetism, which is split into two or more quantized energy levels. Absorption of electromagnetic radiation of appropriate frequency can cause a transition between the generated magnetically-induced energy levels. In a magnetic field, this nuclear-magnetic molecule or nucleus absorbs the energy of the two energy levels different from a low-energy state to a high-energy state, and produces a resonance spectrum, which can be used to determine the number, type, and relative position of certain atoms in the molecule [88]. Nuclear magnetic resonance spectroscopy can obtain the carbon skeleton of unknown compounds and the chemical environment of hydrogen atoms connected to carbon atoms.

The structure information of the compound can be obtained by MS and NMR methods. After the structure analysis, the screening for the biological activity will be carried out to determine a new type of active compound.

### 2.4. Biological Activity Screening

There are some commonly used methods for screening antimicrobial activity, such as the disc diffusion method, solid culture method, liquid dilution method, and so on [89]. However, the current standards of these methods used in different studies are not the same, so that the current screening standards for antimicrobial activity are not uniform, which lead to the mining of marine natural products against plant pathogens has not yet achieved high throughput. The following section will summarize the current screening methods for antimicrobial activity.

#### 2.4.1. Solid Culture Diffusion Method

This is a general method for testing the activity of compounds to plant pathogens [90], by which we can directly observe the effects on the growth of fungi and bacteria. For testing the influence of fungal growth, the mycelial block of the target fungus is placed in the middle of the solid medium, at the same time, a circular paper added with the testing compound is placed not far away from the mycelial block and start to culture. After cultivation for a period of time, the anti-fungal effect can be observed and calculated by measuring the diameter of fungal colonies [91]. This method was used to test the antifungal activity of the compounds towards *A. alternata* and *Phytohthora parasitica* [92]. For testing influence of the bacterial growth, bacteria were coated on the solid agar plate, then four small holes were dug in the plate and, finally, the testing compounds were put into the small holes to observe inhibition effect [93].

The solid culture diffusion method is simple, convenient, and less expensive, and does not require a high amount of compounds. In another similar method, the testing compounds are mixed in an agar medium, and the fungus mycelial block is placed in the agar plate for culture, finally the fungal colony diameter is compared with the control medium without compounds to calculate antimicrobial activity [94]. However, the amount of compounds used in this method is relatively large, lead to limit use.

#### 2.4.2. Dilution Method

Dilution method is the most commonly used method to determine the minimum inhibitory concentration of plant pathogens. This method can simply determine the most effective concentration of antimicrobial compounds to be tested. At present, there are two commonly used dilution methods, one is the liquid culture method, and the other is the solid culture method.

##### Liquid Culture Dilution Method

Liquid culture dilution method can be carried out in 96-well plate, and the amount of compounds to be tested does not need too much. Therefore, this method can not only determine the MIC of the testing compounds, but also can screen the effective compounds against plant pathogens with large flux [95]. Li et al. analyzed the effects of cyclo-(l-leucyl-trans-4-hydroxy-l-prolil-d-leucyl-trans-4-hydroxy-l-proline) on inhibiting of plant pathogenic fungi by liquid culture dilution method [96]. Huang et al. also used this method to confirm the effects of two new cyclic tetrapeptides on the growth of plant pathogenic bacteria [97].

##### Solid Culture Dilution Method

For the solid culture dilution method, the gradient dilute testing compounds are separately added to the culture medium and spread evenly, then flip the plates. The testing plant pathogen is placed in the middle of the plate to determine the antimicrobial effect of the compounds [98]. MIC determination is to detect the concentration of compounds that completely inhibit plant pathogens, and then calculate the MIC value of antimicrobial. For example, Cao et al. used the solid culture dilution method to test the antibacterial effect of six kinds of azaphilone derivatives on *A. brassicola* and *Fusarium oxysporum* [99].

#### 2.4.3. Living Plant Detection

For the living plant detection method, either the testing pathogen or the plant is pre-treated with the compound, and the pathogen is then inoculated to the host plant to observe its invasion capacity. This method can directly determine whether the compounds affect the invasion of plant pathogens, the ultimate goal of screening antimicrobial compounds. Iturin A, a kind of cyclic lipopeptide, was successfully separated from the marine bacterium *Bacillus velezensis*. Iturin A was found to significantly inhibit the rice blast fungus *Magnaporthe oryzae* [100].

### 2.5. Active Substance Mechanism Research

After obtaining a compound with antibacterial activity, we need to study its active mechanism. There are many methods that can be used to clarify the activity mechanism of compounds, and we will review these methods below.

#### 2.5.1. Microscopic Observation

If the plant pathogens were inhibited by an active compound, their cell morphology will usually be affected. Therefore, the changes in the cell morphology of the plant pathogen after the compound treatment can be observed with a microscope to determine the manner in which the compound affects the plant pathogen. For example, using transmission electron microscopy (TEM) and scanning electron microscopy (SEM), the morphology of *M. oryzae* cell walls were found to be severely destroy by fengycin BS155 treatment [77]. Therefore, it can be inferred that the possible target of fengycin BS155 is located in the cell wall or is related to cell wall synthesis. Through microscopic observation, we can preliminarily determine the site of action of the active compound on plant pathogens, laying the foundation for the next in-depth study of the mechanism.

#### 2.5.2. Dyeing Methods

Cell morphology can also be observed by staining methods, such as using Hoechst 33258 to stain chromatin condensation [101], so as to detect whether the compound affects the chromosome morphology. Additionally, JC-1 staining can detect the integrity of the mitochondrial membrane [102], so it can detect whether the compound affects the integrity of the mitochondrial membrane of plant pathogens. The staining method is a supplement to the microscopic observation method, and it can also preliminarily judge the action site of the compound plant pathogenic bacteria.

#### 2.5.3. Omics Methods

After treating plant pathogens with active compounds, transcriptomic, proteomic, or metabonomic analysis can be performed to analyze the specific pathways through which the compounds affect plant pathogens, so that the ways in which the compounds affect plant pathogens can be clearly deduced. For example, an NMR-based metabolome method was used to find that the 2-methyl-N-(2′-phenylethyl)-butanamide and 3-methyl-N-(2′-phenylethyl)-butanamide can affect QS system of the rice pathogenic bacteria Burkholderia glumae’s to disrupt the bacterial communication, thereby inhibiting the growth of pathogenic bacteria [23].

#### 2.5.4. Molecular Docking Methods

**Figure 2:**
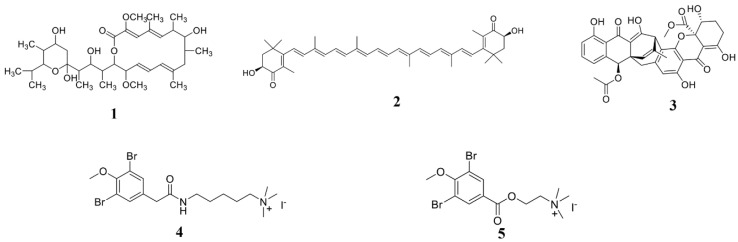
Chemical structures of the compounds **1**–**5**. **1** Bafilomycin A1, **2** astaxanthin, **3** polyketide JBIR-99, and **4**, **5** pulmonarin derivatives compounds 6a and 6b.

Using computer-simulated molecular docking methods, it is possible to predict the interaction between the compounds and the proteins, and then predict the targets of the compounds in the plant pathogens [103]. The acquisition of the targets can clarify the action mechanism of the compounds. However, the molecular docking is only a prediction, therefore, the interaction between the compound and the target requires further biological strategies to verify it. Through a computer-simulated molecular docking method, it was predicted that two pulmonarin derivatives compounds 6a (**4**) and 6b (**5**) could interact with the TMV coat protein (CP), so it can be inferred that compounds **4** and **5** (Figure 2) may inhibit the assembly of TMV particles [104].

## 3. Active Substances Derived from Different Marine Sources

At present, studies have isolated natural products with anti-tumor, anti-microbiol, and other biological activities from marine organisms [12,13,14,16,17,25]. Modern pharmacological research also shows that many marine biological metabolites are effective in killing phytopathogenic microbes and can be used for the development of biological microbicides.

### 3.1. Marine Bacterium-Derived Anti-Microbial Compounds

**Figure 3:**
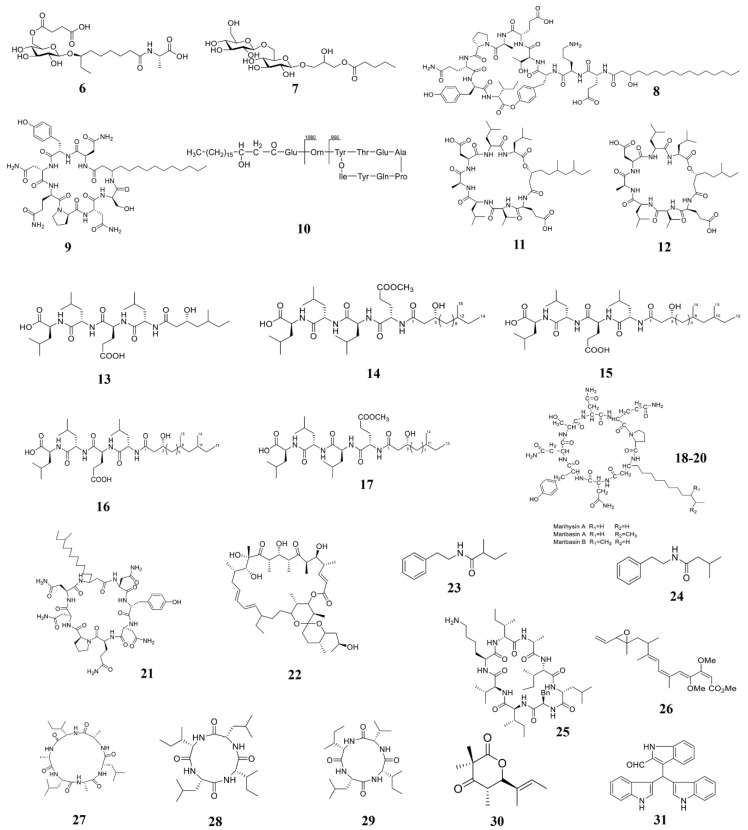
Chemical structures of the compounds identified from marine bacteria. **6** Ieodoglucomide C, **7** ieodoglycolipid, **8** plipastatin A1, **9** iturin A, **10** Fengycin BS155, **11**–**12** gageopeptins A and B, **13**–**16** gageopeptides A–D, **17** gageotetrin B, **18**–**20** Lipopeptides, **21** mojavensin A, **22** oligomycin A, **23** 2-methyl-N-(2′-phenylethyl)-butanamide, **24** 3-methyl-N-(2′-phenylethyl)-butanamide, **25** Champacyclin, **26** Haliangicin, **27**–**29** Halolitoralin, **30** helicascolide C, **31** Trisindolal.

Marine bacteria have great potential to produce new biologically active substances due to their special living environment and metabolic mechanism [12]. Along with the marine microbial research, more and more natural products containing activities of inhibiting plant pathogenic pathogens were identified and separated, many of which obtain novel structures (Table 1; Figure 3).

#### 3.1.1. Active Substances from Marine Bacillus

**Figure 4:**
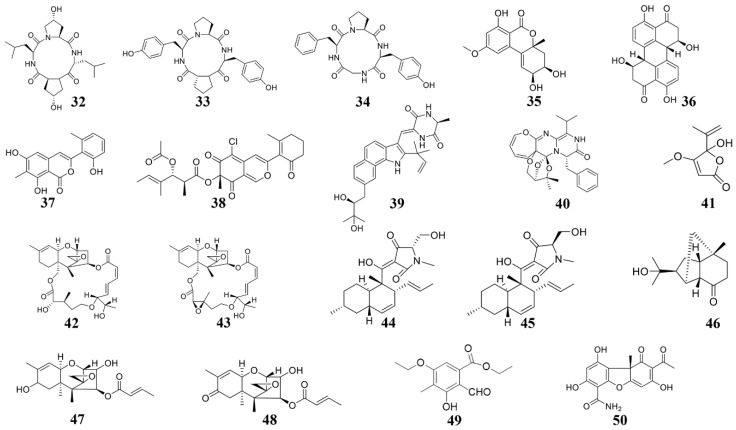
Chemical structures of the compounds identified from marine fungi. **32** Cyclo-(L-leucyl-*trans*-4-hydroxy-L-prolyl-D-leucyl-*trans*-4-hydroxy-L-proline), **33** cyclo (D-Pro-L-Tyr-L-Pro-L-Tyr), **34** cyclo (Gly-L-Phe-L-Pro-L-Tyr), **35** Benzopyranone, **36** Stemphyperylenol, **37** pleosporalone A, **38** pleosporalones B, **39** rubrumazine B, **40** Varioxepine A, **41** Penicillic acid, **42** roridin A, **43** roridin D, **44** equisetin, **45** epi-equisetin, **46**–**48** sesquiterpenes, **49** ethyl 5-ethoxy-2-formyl-3-hydroxy-4-methylbenzoate, **50** (-)-cercosporamide.

*Bacillus* is an important part of marine ecology, which is widely distributed and varied, many important marine natural products were separated from it [119]. Two new glycolipid compounds ieodoglucomide C (**6**) and ieodoglycolipid (**7**), which have antifungal activity against the plant pathogenic fungi, were isolated from the fermentation broth of marine *Bacillus licheniformis* [105]. *Bacillus* of marine origin can secrete some lipopeptide substances, which can be also used to kill plant pathogens. Another compound plipastatin A1 (**8**), was identified from *B. amyloliquefaciens*, which can directly inhibit the germination of gray mold *B. cinerea* [78]. Therefore, compound **8** is a potential sustainable fungicide for *B. cinerea* control. Ma and his collaborators also extracted a cyclic lipopeptide (CLP)-CLP iturin A (**9**) from another strain of *B. velezensis* 11-5 (Figure 4). In vitro experiments showed that compound **9** inhibits rice blast fungus germination and appressorium formation at 10 and 50 µM at 12 hours post-inoculation (hpi) and 24 (hpi). In vivo inoculation experiments showed that compound **9** significantly reduces the ability of rice blast fungus to infect rice at a concentration higher than 10 μM [106]. Therefore, iturin A is another a potential fungicide for fungal disease control. Zhang and Sun isolated the third type of cyclic lipopeptide-fengycin BS155 (**10**) from *B. subtilis* BS155. Morphological experiments showed that fengycin treatment causes mycelial morphological changes of cell membrane and cell wall in the rice blast fungus. It also decreases the mitochondrial membrane potential to cause accumulation of reactive oxygen species (ROS) that results in cytotoxicity [77]. Tareq et al. isolated two new cyclic lipopeptides, gageopeptins A (**11**) and B (**12**) from a marine-derived bacterium *Bacillus* sp. 109GGC020. Compounds **11** and **12** significantly inhibited the growth of *P. capsica*, *R. solani*, and *B. cinerea* [107]. Later, Chakraborty et al. also isolated and extracted five lipopeptides (**13**–**17**) from *B. subtilis* 109GGC020. Compounds **13**–**17** all inhibit the hyphal growth of *M. oryzae* Triticum (MoT) [24]. Gu et al. also isolated and extracted some lipopeptides (**18**–**20**) from the marine source *Bacillus marinus* B-9987. Compounds **18**–**20** have been shown to have the activity to kill *B. cinerea* both in vitro and in vivo [108]. Ma et al. isolated a new iturinic lipopeptide, mojavensin A (**21**) from *Bacillus mojavensis* B0621A. In vitro anti-fungal activity test showed that compound **21** exhibited dose-dependent antifungal activity against *V. mali*, *F. oxysporum* f. sp. *cucumerinum*, and *F. verticillioides* [109]. Therefore, lipopeptides are common phytopathogenic compounds in marine *Bacillus*. Besides, *Bacillus* of marine origin also produces some chitinases, which also have the activity of killing plant pathogenic fungi. Bhattacharya et al. isolated and extracted a heat-resistant and alkali-resistant chitinase from *B. pumilus* JUBCH08, which has a strong *F. oxysporum* inhibitory activity [110]. 

#### 3.1.2. Active Substances from Marine Streptomyces

Marine *Streptomyces* is another important natural product source of marine microbes. Studies have shown that the most widely studied marine actinomycetes are *Streptomyces*, which accounts for 60% of the new natural products of marine actinomycetes [120]. However, according to natural products to kill the plant pathogens, there are relatively less reports on that derived from *Streptomyces*. In marine mud, Long et al. isolated two *Streptomyces* strains, *S. roseobiolascens* XAS585 and *S. roseofulvus* XAS588, whose fermentation broth inhibit spore germination of the plant pathogen *A. alternate.* This result suggested that *S. roseobiolascens* XAS585 and *S. roseofulvus* XAS588 contain microbiocidal substances required for further separation and extraction [111]. Buatong et al. isolated and extracted a compound oligomycin A (**22**) from the marine *Streptomyces* sp. Compound **22** breaks the fungal cell membrane and organelles, and inhibit spore germination and appressorium formation [118], makes it a promising biological fungicide. Recently, two compounds 2-methyl-N-(2’-phenylethyl)-butanamide (**23**) and 3-methyl-N-(2’-phenylethyl)-butanamide (**24**) were extracted from the marine *Streptomyces* sp. PNM-9, both compounds showed the activity of killing the rice pathogen *B. glumae*, suggesting they could be used as potential biological fungicides [23]. Pesic et al. isolated a new cyclic octapeptide, champacyclin (**25**), from *Streptomyces* strain C42, which showed antimicrobial activity against *E. amylovora* [112].

#### 3.1.3. Active substances from other marine bacteria

Other types of bacteria may also produce compounds with microbicide activity towards phytopathogenic pathogens. Fudou et al. isolated and extracted a new type of β-methoxyacrylate compound-haliangicin (**26**) from the culture medium of marine slime mold. Compound **26** shows broad-spectrum fungicidal activity, but no bactericidal activity [113]. Yang et al. isolated and extracted several cyclic peptides (**27**–**29**) from the marine bacterium *Halobacillus litoralis* YS3106. Compounds **27**–**29** obtain simple structures and highly repetitive residue units, which show moderate antifungal activity against plant pathogens [114]. Manwar et al. isolated pyoverdine type of siderophores from fluorescent *Pseudomonas* sp. under low iron stress. This type of siderophores shows high antifungal activity [115]. From the endogenous *Daldinia eschscholzii* isolate of the red alga, Tarman et al. extracted a compound helicascolide C (**30**), which shows high fungicidal activity against the plant pathogen *C. cucumerinum*. Nair et al. isolated and extracted a new indole alkaloid compound trisindolal (**31**) from *Vibrio splendidus* T262, which significantly inhibits *B. cinerea* and *P. infestans* [116].

### 3.2. Marine Fungus-Derived Anti-Microbial Compounds

Natural products derived from marine fungi are one of the most important sources of pharmaceutical lead compounds, most of which are widely identified for use in medicine, agriculture, and other fields due to their bioactive activity [121]. Among known marine-derived compounds used for killing plant pathogens, that derived from marine fungi is the largest ones (Table 2; Figure 4).

#### 3.2.1. Active Substances from Marine Alternaria

In 2014, several cyclic tetrapeptides were extracted from the medium co-culturing *Phomopsis* sp. K38 and *Alternaria* sp. E33, two mangrove fungi [96,122]. Among these cyclic tetrapeptides, cyclo-(L-leucyl-trans-4-hydroxy-L-prolyl-D-leucyl-trans-4-hydroxy-L-proline) (**32**) is the most effective for suppressing the plant fungal pathogens [122]. The cyclo (D-Pro-L-Tyr-L-Pro-L-Tyr) (**33**) and cyclo (Gly-L-Phe-L-Pro-L-Tyr) (**34**) both showed inhibiting effects on the plant pathogens such as *G. graminis* and *F. graminearum* [96]. These cyclic tetrapeptides can be considered for developing new fungicides. Additionally, in 2018, from more than 100 strains of marine fungi, two fungi, *Fusarium equiseti* (P18) and *Alternaria* sp. (P8), which show microbicidal activity against plant pathogens were identified. Subsequently, several active substances were extracted from these two fungi [123,128]. Among these, benzopyranone (**35**) and stemphyperylenol (**36**) suppress the growth of *A. brassicicola* [123,128].

#### 3.2.2. Active Substances from Marine Pleosporales

*Pleosporales* sp. CF09-1, a fungus isolated from the Bohai Sea region of China, was used to extracted some active substances, including pleosporalone A (**37**) and pleosporalone B (**38**) [99]. Pleosporalone A shows strong fungicidal activity against the plant fungal pathogens such as *B. cinerea* [99]. Pleosporalone B also shows strong fungicidal activity against the plant pathogens such as *F. oxysporum* [99]. Therefore, pleosporalone A and pleosporalone B possess great potential to be developed as biological pesticides.

#### 3.2.3. Active Substances from Marine Parasitic Fungus

Many marine fungi live on a certain substrate, and the special parasitic relationship of these fungi can produce natural products with new functions and new structures [130]. Du et al. extracted 13 indomethacin alkaloid compounds from *Eurotium cristatum* EN-220 [125], among which, rubrumazine B (**39**) shows a certain blasticidal activity [125]. A 3H-Oxepine-containing alkaloid compound varioxepine A (**40**) was also extracted from another fungus *Paecilomyces variotii* isolated from marine algae. Varioxepine A inhibits the growth of *F. graminearum* [126]. Huang et al. extracted a compound penicillic acid (**41**) from the *Aspergillus* sp. D40, which was isolated from seaweed. Penicillic acid broadly inhibit plant bacteria such as *R. solanacearum*, *Xanthomonas campestris*, and *X. citri* [22]. Additionally, there are many compounds with novel structures in sponge fungi. For example, Xie et al. extricated roridin A (**42**) and roridin D (**43**), two macrocyclic trichothecene compounds from *Myrothecium* sp. fungus disconnected from sponge *Axinella* sp [127]. Roridin A and roridin D severely inhibit fungal pathogens such as *M. oryzae* and *S. sclerotiorum* [127].

#### 3.2.4. Active Substances from Other Marine Fungus

Zhao et al. extracted two compounds equisetin (**44**) and epi-equisetin (**45**) from *F. equiseti* D39, a marine fungus. Equisetin (**44**) and epi-equisetin (**45**) exhibited striking antibacterial activities to *P. syringae* and *R. cerealis* [123]. A number of compounds, including three compounds (**46**–**48**) showing antifungal activities against *C. lagrnarium* and *B. cinerea*, were extracted from the marine fungus *Trichoderma longibrachiatum* [21]. From the marine fungus *Nos*. K38 and E33 co-culture medium, a new polysubstituted benzaldehyde compound, ethyl 5-ethoxy-2-formyl-3-hydroxy-4-methylbenzoate (**49**), was extracted. This compound broadly inhibits the growth of plant pathogens such as *R. solani*, *F. graminearum*, and *P. sojae* [129], therefore could be developed as a broad-spectrum fungicide. The compound (-)-cercosporamide (**50**) was extracted from another marine fungus *Verruculina enalia* BCC 22226, which shows antifungal activity towards *M. oryzae* and *C. acutatum* [20] and makes compound **50** a candidate for developing antifungal agents.

### 3.3. Sponge-Derived Anti-Microbial Compounds

**Figure 5:**
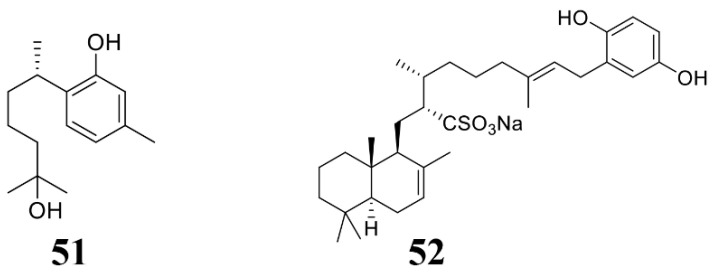
Chemical structures of the compounds identified from marine sponges. **51** (+)-Curcuphenol, **52** halisulfate 1.

Sponges are the most primitive multicellular animals, which live in different environments in the ocean, making them rich in active substances. At present, sponges have also become an important resource for the extraction of marine-derived antimicrobial compounds (Table 3; Figure 5) [66]. For example, Gaspar et al. extracted a metabolite from Caribbean sponge *Didiscus oxeata*, (+)-curcuphenol (**51**), which inhibited the growth of a large number of plant fungal pathogens, inlcuding *B. cinerea* and *F. oxysporum* (Table 3) [131]. Shin et al. isolated a sulfate and isocitrate lyase (ICL) inhibitor halisulfate 1 (**52**) from the tropical sponge *Hippospongia* spp. Halisulfate 1 can reduce the appressorium formation of the blast fungus, thereby suppress infection of the blast fungus. Moreover, the infection phenotype of *M. oryzae* treated with halisulfate 1 is consistent with that of *M. oryzae* Δ*icl* knockout mutant, suggesting that halisulfate 1 may act on isocitrate lyase [132], and detailed mechanism is not known.

### 3.4. Seaweed-Derived Anti-Microbial Compounds

**Figure 6:**
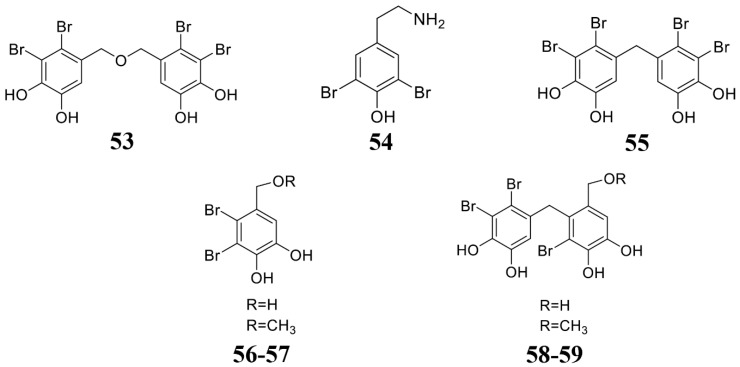
Chemical structures of the compounds identified from marine seaweeds. **53** Bis(2,3-dibromo-4,5-dihydroxybenzyl) ether (BDDE), **54**–**59** bromophenols.

Seaweed is considered as a plant that grows in the ocean. Due to its special growth environment, it also produces rich active substances (Table 4; Figure 6). Liu et al. found that bis(2,3-dibromo-4,5-dihydroxybenzyl) ether (BDDE) (**53**) extracted from seaweed significantly inhibits the growth of gray mold either on solid PDA or liquid medium PDB. Additionally, treatment by compound **53** can significantly reduce the incidence of strawberry fruit rot and gray mold. Mechanism studies reveal that compound **53** destroys the integrity of gray mold spores and newly formed germ tube cell membranes [133]. Lee et al. isolated a class of isocitrate lyase inhibitor bromophenol compounds (**54**–**59**) from the red alga *Odonthalia corymbifera*. Compounds **54**–**59** inhibit the activity of ICL and prevent the appressorium formation of *M. oryzae*, thereby reducing its infection [134]. Felício et al. obtained two fractions n-hexane (BT-H) and dichloromethane (BT-D) from the extract of the red algae *Bostrychia tenella* J. Agardh. These two fractions can inhibit the growth of *Cladosporium cladosporioides* and *Cladosporium sphaerospermum* [135]. Seaweed is rich in polysaccharides, which also show inhibiting activity to the plant pathogenic pathogens [136]. Righini et al. extracted polysaccharides from *Ecklonia* sp., *Anabaena* sp., and *Jania* sp., which inhibit the growth of gray mold [137]. The microalgal phenolic extracts (MPE) were obtained from *Nannochloropsis* sp. and *Spirulina* sp. These MPE extracts significantly inhibited fungal growth [138]. All the above shows that microalgae phenolic substances are also an important source for developing phytopathogenic fungicides.

### 3.5. Marine Animal-Derived Anti-Microbial Compounds

Marine animals may also become an important source to extract marine natural products, but there are fewer studies on anti-microbiol compounds derived from marine animals (Table 5). From the marine snail *Cenchritis muricatus* López-Abarrategui et al. isolated a 1487.26 Da peptide Cm-p1 in the crude extract. Cm-p1 can evidently inhibit the growth of *F. oxysporum* and *B. cinerea* [140]. Similarly, Wang et al. found a hepcidin gene in large yellow croaker (*Pseudosciaena crocea*), which is expressed to produce antimicrobial peptides. This antimicrobial peptide can evidently inhibit the growth of plant pathogens such as *F. graminearum* [141]. These results suggest that marine animals also produce antimicrobial substances, which have broad-spectrum antibacterial activity.

## 4. Microbicidal Mechanisms of Marine-Derived Bioactive Substances

**Figure 7:**
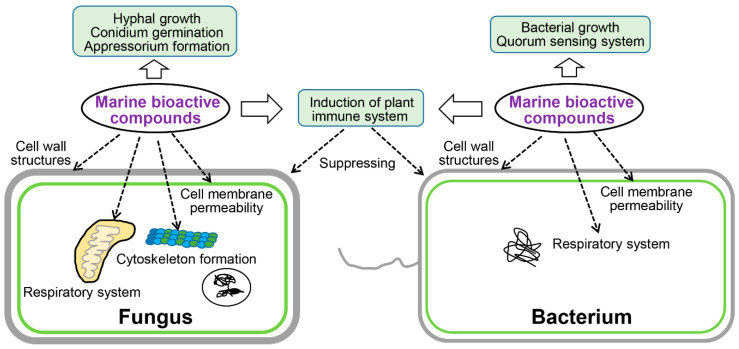
Mechanism of action of active substances identified from marine sources.

Marine natural compounds suppress or kill plant pathogenic pathogens through different mechanisms, and the reported antimicrobial mechanisms of marine natural compounds are summarized in Figure 7, including affecting microbial cell wall synthesis, cell membrane permeability, fatty acid metabolism, respiratory system, cytoskeleton, bacterial quorum sensing (QS), as well as inducing plant immune system for inhibition.

### 4.1. Affect Cell Wall Structures

Cell wall is the outermost structure of the plant pathogenic fungi and bacteria, which plays important role in maintaining cell shape and integrity. It also maintains normal metabolism, ion exchange, and osmotic pressure in cells [142]. Some marine compounds can inhibit the formation of microbial cell walls, thus suppress the growth or kill the pathogens. For instance, Chakraborty et al. isolated compounds **13**–**17** from a marine bacterium *B. subtilis* 109GGC020. Compounds **13**–**17** inhibit cell wall synthesis of *M. oryzae* to inhibit its growth [24]. Marine natural products can destroy structure of the fungal cell wall component glucosamine to inhibit the cell wall synthesis and growth. For example, microalgal phenolic extracts (MPE) were isolated from marine microalgae *Nannochloropsis* sp. and *Spirulina* sp., which can destroy the glucosamine structure of *F. graminearum* CQ244 and reduce glucosamine production by 15% [138]. Chitin is another fungal cell wall key component, which can be degraded by chitinase [143]. A chitinase was identified from the marine bacterium *B. pumilus* JUBCH08, which was proved to degrade the cell wall of *F. oxysporum* and inhibit its growth [109].

### 4.2. Affect Cell Membrane Permeability

The cell membrane is a lipid bilayer semi-permeable membrane, which controls the two-way flow of substances inside and outside the cell of the plant pathogens. The microbial cell membrane is another most common target of marine natural compounds. For example, antifungal ethyl acetate extract from marine-derived *Streptomyces* sp. AMA49 can destroy the cell membrane of *M. oryzae* to suppress fungal growth [118]. A series of other marine-derived natural compounds can also achieve their antibacterial effects by destroying the cell membrane of different plant pathogens [77,123,128,133,140,141]. Compound **9** isolated from *B. velezensis* 11-5 can selectively bind with phospholipids in the cell membrane of *M. oryzae*, thus affecting the membrane structure [106]. The microalgal phenolic extracts, which are isolated from *Nanochoropsis* and *Spirulina*, can combine with ergosterol on the membrane (MPE) of *F. graminearum* cells to elevate plasma membrane permeability and lead to the leakage of proteins, nucleotides, amino acids, sugars, and salts, therefore leading to the death of fungi [139]. 

### 4.3. Affect Fatty Acid Metabolism

Fatty acid metabolism is very important for the functional appressorium formation in some plant pathogenic fungi. When the fatty acid metabolism of fungi is blocked, the fungi cannot penetrate plant cells [144,145,146]. Interestingly, it is reported that halisulfate 1 and bromophenols, the marine-derived metabolites, could inhibit the activity of *M. oryzae* isocitrate lyase, thus inhibiting the fatty acid metabolism, which will affect mature appressorium formation and penetration of *M. oryzae* [132,147].

### 4.4. Affect Respiratory System

Marine natural compounds can also target the respiratory electron transport system of plant pathogens to inhibit their respiration, through which they can inhibit the growth of pathogens. For example, compound **26** (Figure 3) isolated from marine *Myxobacterium* can breaking the electron flow by targeting the cytochrome b-c1 segment [113], and therefore inhibits the growth of plant pathogens.

### 4.5. Affect Cytoskeleton Formation 

The cytoskeleton also plays important roles for fungal development and infection processes [148], whose components could be bound by the marine compounds and inhibit fungal growth and infection. For instance, the marine organisms derived compounds **46**–**48** (Figure 4) can inhibit the growth of *Colletotrichum* species and *B. cinerea* [21], the mechanism is that compounds **46**–**48** target the fungal β-tubulin proteins and subsequently suppress cell division [21].

### 4.6. Affect Bacterial QS System

QS system can help bacteria to monitor the quantity change of itself or other bacteria in the surrounding environment, according to the concentration change of specific signal molecule autoinducer. When the signal molecule reaches a certain concentration threshold, it can start the expression of related genes in the bacteria to adapt to the environmental changes. When the QS system of bacteria is blocked, bacteria cannot communicate with the surrounding environment and failed to infect the host plant. Interestingly, some marine natural compounds can also target and interrupt the bacterial QS system to prevent infection of the bacterial pathogens [149,150]. For example, 2-methyl-n-(2’-phenylethyl)-butanamide and 3-methyl-n-(2’-phenylethyl)-butanamide, two marine-derived compounds, can destroy the QS system of rice pathogenic bacterium *B. glumae* (ATCC 333,617), therefore interrupt virulence feature production, including proteases, toxins, as well as some other immune-evasion factors [23]. In this situation, the QS signal is blocked by the marine compounds, and the bacteria fail to attack the host.

### 4.7. Induction of the Plant Immune System

**Figure 8:**
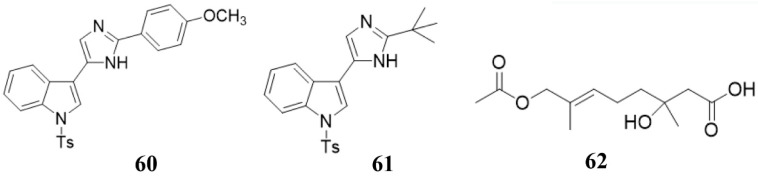
Chemical structures of the compounds **60** (nortopsentin alkaloid 2e), **61** (nortopsentin alkaloid 2k), and **62** penicimonoterpene (±).

Some marine natural products can not directly inhibit or kill bacteria against plant pathogens, but be used as elicitors to stimulate the plant immune system to inhibit or kill bacteria, which serve as an indirect antibacterial or bactericidal effect [151]. Ji et al. and Righini et al. isolated a series of compounds (**60**, **61**, polysaccharides) from marine organisms (Figure 8) [137,152]. The activity test of these compounds showed that they could not directly inhibit the growth of plant pathogens, but stimulate the plants to resist to plant pathogens [137,152]. This elegant work clearly showed that the marine compounds could indirectly inhibit plant pathogens by inducing the host immune system.

## 5. Potential of Large-Scale Application of Marine Natural Products in Agricultural Production

In the previous section, we summarized marine bioactive antimicrobial compounds for plant pathogens. Some of them have particularly good activity and have great potential as new biopesticides against plant pathogens in the future. For example, compounds **6** and **7** isolated from *B. licheniformis* had MIC values of only 0.03–0.05 μM against plant pathogenic fungi [105]; while compound **38** isolated from *Pleosporales* sp. CF09-1, whose MIC values against plant pathogens *A. brassicicola* and *F. oxysporum* are also only 1.6 μg/mL [99]. Nevertheless, the large-scale application of these compounds still has a long way to go. This is because the yield of these compounds from marine organisms is relatively low, which limits the large-scale use of these compounds. In order to meet the large-scale use of these compounds, there are currently two methods, one is a chemical synthesis method, and the other is a synthetic biology method.

### 5.1. Chemical Synthesis Methods

For some compounds whose content is low and whose yield cannot meet people’s needs, it is necessary to increase the yield of compounds through chemical synthesis methods [153]. The starting compound of chemical synthesis is a compound that is relatively easy to obtain and contains a large amount. Then, through a series of physical or chemical methods, a sufficient amount of the target compound is finally obtained [153]. Compared with the natural product yield in micrograms, the product can be obtained in grams using the chemical synthesis method [154]. In addition to improving yield, chemical synthesis can also modify active compounds to obtain compounds with higher activity [154]. This greatly increases the possibility of large-scale application of the compound.

Wang’s lab has done a lot of work in this area [104,126,152]. In 2014, they fully synthesized penicimonoterpene (±) (**62**) (Figure 7) for the first time using a Reformatsky reaction, which started from the 6-methylhept-5-en-2-one precursor compound and the yield of the entire synthesis process was 25–85% through a four-step reaction [126]. Additionally, using similar synthetic methods, a series of compound **62** derivatives were also obtained. These synthesized compounds not only increased the yield, but also increased their resistance to plant pathogens [126]. For example, some compounds showed extremely high selective against plant-pathogenic fungus *F. graminearum* (MIC 0.25 μg/mL) [126]. In 2018, they modified the active substance nortopsentin alkaloids and obtained a series of nortopsentin derivatives [152]. The activity of these derivatives against the plant pathogenic virus TMV was significantly improved compared with pre-modification [152]. Recently, they have effectively synthesized the compounds **4** and **5** (pulmonarins A and B, Figure 2) through a total synthesis method, with yields as high as 64% and 59%, respectively [104]. In addition, compounds **4** and **5** have high resistance to TMV [104]. These results all indicate that chemical synthesis can greatly increase the yield and activity of active compounds, making it possible for these compounds to be applied on a large scale in agriculture.

### 5.2. Synthetic Biology Methods

Synthetic biology is currently a hot field in the field of biology. It can make compounds that are difficult to synthesize or have low yields in the body to be heterologously expressed in engineered microorganisms, which can enable the synthesis of a large number of target compounds and increase the yield [155]. In the field of biomedicine, synthetic biology has been greatly developed [155]. For example, the genes that synthesizes paclitaxel and artemisinin could be expressed in yeast, so that paclitaxel and artemisinin can be extracted from yeast, which not only improves paclitaxel and artemisinin yield, but also reduces the cost of producing them [156,157]. Additionally, with the reduction of sequencing costs, more and more biological genomes have been sequenced, which enables scientists to dig out gene clusters that can produce new compounds from more genomes, thereby promoting the development of natural products field [158]. The current development of artificial intelligence technology has also made it possible to synthesize more active compounds [159]. In short, synthetic biology represents the future of natural products.

## 6. Prospects and Challenges

Reducing the impact of plant diseases is extremely important for food security. Traditional chemical agents can significantly kill plant pathogenic fungi and bacteria, but they are limited in use due to problems such as pathogen resistance, non-degradability, environmental safety, and human health. Therefore, the development of environmentally-friendly biological microbicides will become urgent. With the continuous decrease of terrestrial resources, marine resources have gradually become a research focus for scientists. Some valuable marine-derived active substances have been identified or effectively used in plant disease control. However, there are still some problems in this field. First of all, the marine-derived compound numbers used in the control of the plant pathogens is much less than that of the human pathogens. More attention should be paid by researchers to discover more active and better marine-derived compounds used in the control of the plant pathogens. Secondly, the current research on the application of marine-derived active compounds in the prevention and control of plant pathogenic microbes is only to detect the microbicidal activity of the compound. The study on the mechanism of their microbicidal activity and their targets, which can enable people to better understand plant pathogens and provide a theoretical basis for the later artificial synthesis of specific compounds is far more than enough. Thirdly, at present, there is almost no application of synthetic biology in the field of marine-derived active substances against plant pathogens. More people who have both sufficient chemical knowledge and a biological background need to be engaged in this field. Finally, current activity screening of the marine-derived compounds is not suitable for a large-scale experiment, which significantly limits the study. A new high-throughput screening platform is urgent to build for quick and large-scale discovery of new marine active compounds.

The abundant marine resources of the Earth provide broad space for research and development in this field. With the improvement of screen strategies and biotechnologies, combining with the synthetic biology techniques, novel active marine-derived compounds will be isolated and utilized in the prevention and control of plant pathogens, to increase food production and guarantee human food security.

## Figures and Tables

**Table 1:** Recent studies in the screening of active substances from marine bacteria.

Bacteria Sources	Substances	Activity to Pathogens	Year [Ref]
*B. licheniformis* 09IDYM23	Ieodoglucomide C (**6**) and ieodoglycolipid (**7**)	*Aspergillus niger*, *Botrytis cinerea*, *Colletotrichum acutatum*, *R. solani*	2015 [105]
*B. amyloliquefaciens* SH-B74	Plipastatin A1 (**8**)	*B. cinerea*	2018 [78]
*B. velezensis* 11-5	Iturin A (**9**)	*M. oryzae*	2019 [106]
*B.s subtilis* BS155	Fengycin BS155 (**10**)	*M. oryzae*	2018 [77]
*Bacillus* sp. 109GGC020	Gageopeptins A (**11**) and B (**12**)	*P. capsica*, *B. cinera*, *R. solani*	2015 [107]
*B. subtilis* 109GGC020	Gageopeptides A–D (**13**–**16**) and gageotetrin B (**17**)	*M. oryzae* Triticum (MoT)	2020 [24]
*B. marinus* B-9987	Lipopeptides (**18**–**20**)	*B. cinerea*	2017 [108]
*B. mojavensis* B0621A	Mojavensin A (**21**)	*Valsa mali*, *F. verticillioides*, *F. oxysporum*	2012 [109]
*B. pumilus* JUBCH08	Chitinase	*F. oxysporum*	2016 [110]
*S. roseobiolascens* XAS585, *S. roseofulvus* XAS588	Fermented broth (FBE)	inhibit mycelial development and conidial germination	2011 [111]
*Streptomyces* sp. AMA49	Oligomycin A	*Pyricularia oryzae*	2019 [112]
*Streptomyces* sp. PNM-9	2-methyl-N-(2′-phenylethyl)-butanamide (**23**) 3-methyl-N-(2′-phenylethyl)-butanamide (**24**)	*B. glumae*	2020 [23]
*Streptomyces* Strain C42	Champacyclin (**25**)	*Erwinia amylovora*	2013 [113]
*Haliangium luteum* AJ-13395	Haliangicin (**26**)	a wide range of fungi	2001 [114]
*H. litoralis* YS3106	Halolitoralin (**27**–**29**)	moderate antifungal activity	2002 [115]
*Pseudomonas aeruginosa*	Siderophores	*A. niger*, *A. oryzae*, *A. flavus*, *F. oxysporum*, *Sclerotium rolfsii*	2004 [116]
*Daldinia eschscholzii*	Helicascolide C (**30**)	*Cladosporium cucumerinum*	2012 [117]
*Vibrio splendidus* T262	Trisindolal (**31**)	*Phytophthora infestans, B. cinerea*	2016 [118]

**Table 2:** Recent studies in the screening of active substances from marine fungi.

Fungi Sources	Substances	Activity to Pathogens	Year [Ref]
*Phomopsis* sp. K38 and *Alternaria* sp. E33	Cyclo-(L-leucyl-*trans*-4-hydroxy-L-prolyl-D-leucyl-*trans*-4-hydroxy-L-proline) (**32**)	*G. graminis*, *F. graminearum*, *R. cerealis*, *H. sativum*	2014 [96]
*Phomopsis* sp. K38 and *Alternaria* sp. E33	Cyclo (D-Pro-L-Tyr-L-Pro-L-Tyr) (**33**) and cyclo (Gly-L-Phe-L-Pro-L-Tyr) (**34**)	*G. graminis*, *F. graminearum*, *R. cerealis*, *H. sativum*	2014 [122]
*Alternaria* sp. (P8)	Benzopyranone (**35**)	*A. brassicicola*	2018 [84]
*Fusarium equiseti* (P18) and *Alternaria* sp. (P8)	Stemphyperylenol (**36**)	*A. brassicicola, Pestallozzia theae*	2018 [123]
*Pleosporales* sp. CF09-1	Pleosporalone A (**37**)	*B. cinerea*, *Phytophthora capsica, R. oryzae*	2016 [124]
*Pleosporales* sp. CF09-1	Pleosporalones B (**38**)	*F. oxysporum, A. brassicicola*	2019 [99]
*Eurotium cristatum* EN-220	Rubrumazine B (**39**)	*M. oryzae*	2017 [125]
*Paecilomyces variotii*	Varioxepine A (**40**)	*F. graminearum*	2014 [126]
*Aspergillus* sp. D40	Penicillic acid (**41**)	*Ralstonia solanacearum*, a few other bacteria	2020 [22]
*Myrothecium* sp.	Roridin A (**42**) and roridin D (**43**)	*Sclerotinia sclerotiorum, M. oryzae*	2008 [127]
*Fusarium equiseti* D39	Equisetin (**44**) and epi-equisetin (**45**)	*P. syringae*, *R. cerealis*	2019 [128]
*Trichoderma longibrachiatum*	Sesquiterpenes (**46**–**48**)	*B. cinerea, C. lagrnarium*	2020 [21]
*Nos.* K38 and E33	Ethyl 5-ethoxy-2-formyl-3-hydroxy-4-methylbenzoate (**49**)	*F. graminearum*, *R. solani*, *P. sojae, Gloeosporium musae*	2013 [129]
*Verruculina enalia* BCC 22226	(-)-cercosporamide (**50**)	*M. oryzae, C. acutatum*	2020 [20]

**Table 3:** Recent studies in the screening of active substances from marine sponges.

Sponge Sources	Substances	Activity to Pathogens	Year [Ref]
*Didiscus oxeata*	(+)-curcuphenol (**51**)	*C. cucumerinum*, *F. oxysporum*, *A. ramosa*, *A. niger*, *B. cinerea*, *P. expansum*, *R. oryzae*, *T. Harzianum*, *T. mentagrophytes*, *T. Koningii*	2004 [131]
*Hippospongia* spp.	Halisulfate 1 (**52**)	*M. oryzae*.	2007 [132]

**Table 4:** Recent studies in the screening of active substances from marine seaweeds.

Seaweed Sources	Substances	Activity to Pathogens	Year [Ref]
*Leathesia nana*, *Rhodomela confervoides*, *Rhodomela confervoides*	Bis(2,3-dibromo-4,5-dihydroxybenzyl) ether (BDDE) (**53**)	*B. cinerea*.	2014 [133]
*Odonthalia corymbifera*	Bromophenols (**54**–**59**)	*M. oryzae*.	2007 [134]
*Bostrychia tenella* J. Agardh (Rhodomelaceae, Ceramiales)	n-hexane (BT-H) and dichloromethane (BT-D)	*C. sphaerospermum*, *C. cladosporioides*	2010 [135]
*Anabaena* sp., *Ecklonia* sp., *Jania* sp.	Water extracts and polysaccharides	*B. cinerea.*	2019 [137]
*Nannochloropsis* sp. and *Spirulina* sp.	Microalgal phenolic extracts (MPE)	*F. graminearum*	2019 [139]

**Table 5:** Recent studies in the screening of active substances from marine animals.

Marine Animal Sources	Substances	Activity to Pathogens	Year [Ref]
*Cenchritis muricatus*	Cm-p1	*T. rubrum* and *C. neoformans*	2012 [140]
*Pseudosciaena crocea*	PC-hepc	Wide-spectrum against bacteria and fungi	2009 [141]
